# Optimizing textile dyeing wastewater for tomato irrigation through physiochemical, plant nutrient uses and pollution load index of irrigated soil

**DOI:** 10.1038/s41598-022-11558-1

**Published:** 2022-06-16

**Authors:** Jahidul Hassan, Md. Mijanur Rahman Rajib, Umakanta Sarker, Masuma Akter, Md. Noor-E.-Azam Khan, Shahjalal Khandaker, Farhan Khalid, G. K. M. Mustafizur Rahman, Sezai Ercisli, Crina Carmen Muresan, Romina Alina Marc

**Affiliations:** 1 Department of Horticulture, Bangabandhu Sheikh Mujibur Rahman Agricultural University, Gazipur, 1706 Bangladesh; 2 Department of Genetics and Plant Breeding, Bangabandhu Sheikh Mujibur Rahman Agricultural University, Gazipur, 1706 Bangladesh; 3 Department of Textile Engineering, Dhaka University of Engineering and Technology, Gazipur, 1700 Bangladesh; 4 Faculty of Agriculture and Environment, The Islamia University of Bahawalpur, Bahawalpur, Pakistan; 5 Department of Soil Science, Bangabandhu Sheikh Mujibur Rahman Agricultural University, Gazipur, 1706 Bangladesh; 6 Department of Horticulture, Faculty of Agriculture, 25240 Erzurum, Turkey; 7 Food Engineering Department, Faculty of Food Science and Technology, University of Agricultural Sciences and Veterinary Medicine, 400372 Cluj-Napoca, Romania

**Keywords:** Plant sciences, Environmental sciences, Natural hazards

## Abstract

Reuse of wastewater for vegetable cultivation is becoming popular in order to augment the inadequate irrigation supplies and meet the growing demands of ground water for agriculture and industries production in different regions of the world. This study was investigated to optimize different stages of textile dyeing wastewater (TDW) for irrigation focusing on their effect on growth, yield and physiochemical attributes of tomato, plant nutrient use, heavy metals enrichment and pollution load of the irrigated soil. Textile wastewater were collected from the seven stages of (second wash after scouring and bleaching T2; enzyme treated water T3; second wash after bath drain T4; neutralization treatment T5; second wash after soaping T6; fixing treatment water T7; mixed effluent T8) of a dyeing process for physiochemical characterization and evaluation their irrigation feasibility for tomato cultivation in compare with the ground water (T1). The pot experiment consists of eight irrigation treatments was laid out following a completely randomized block design with three replications. Results showed the presence of plant nutrients and heavy metals in all the studied samples where T8 (mixed effluent) exceeded the limit of agricultural standard for almost all physiological parameters such as TDS, TSS, EC, BOD, COD affording the highest value. T8 also delivered the highest Cl- and heavy metals like Cd, Ni, Cr followed by T4 < T7. As a consequence, these provided comparatively higher enrichment factor (EF), pollution load index (PLI) and sodium absorption ratio (SAR) to transform fresh soil into the category of severe and slightly to moderate saline. Therefore, the yield and physiochemical attributes of tomato were dramatically reduced with T8 and T4 treatment. On the other hand, T2, T3 and T6 treatment had significant positive impact on growth and yield of tomato due to having higher N, P, K, S and lower heavy metals (Cu, Zn, Fe, Pb, Cd, Ni, Cr) than the recommended guideline. These features were contributed to cause minimum EF and PLI in the soil irrigated with T2, T3 and T6 stages of TDW. Correlation matrix demonstrated that EF and PLI of heavy metals (except Cd, Ni) were negatively related to yield, while positively related to SAR and fruit abortion. Although T6 (2nd wash after soaping) performed better in respect to growth, yield, yield attributes and nutrient use efficiency, principal component analysis revealed that T2 (2nd wash after scouring and bleaching) and T3 (enzyme treated water) were also belong to the same group of T6 and T1 (ground water). Thus, it may be suggested that T2, T3 and T6 stages of textile dyeing wastewater could be used profitably without ETP for vegetable cultivation and would effectively supplement not only the nutrient requirement of the crop but may also act as the alternate source of irrigation water. Although, further research is needed to sort out the health risk assessment through the heavy metals’ accumulation in the plant parts after irrigation with different stages of textile dyeing wastewater.

## Introduction

The economy of Bangladesh is predominantly based on agriculture that uses about 70% of freshwater resources which is increasing proportionately to population growth and rapid economic developments^1^. Likewise, urbanization as well as industrialization other two driving forces of boosting economy is rushing to consume a huge quantity of water. Bangladesh is the twelfth largest garment manufacturing nation in the world and contributes 77% to the country’s foreign exchange earnings with 50% of its industrial workforce^2^. Among the different industries, textile dyeing is considered to be one of the world’s worst polluters because it uses large quantity of water in its production processes and highly polluted and toxic waste waters are discharged into sewers, drains, agricultural land without any kind of treatment that denoted as wastewater^3^. In the dyeing process, there are 17 steps are involved where the fabrics are passing through two subsequent processes of washing and dyeing^4,5^. In each section, the selected fabric is first washing with fresh water then dyeing and again rinse with the fresh water. The water is discharge in every time after each process of dyeing (Supplementary Fig. 1A). It has been estimated that for dyeing 1 kg of cotton goods with reactive dyes, it requires an average of 70–150 L water, 0.6 kg NaCl, 40 g reactive dyes (organic and inorganic chemicals), alkalis (NaOH) and dyeing auxiliaries^6^. Textile dyeing wastewater (TDW) are being continuously disposal into the surrounding areas causing alteration of the physical, chemical, and biological properties of ecological resources that is considered as the global threat for the sustainable biodiversity^4,5^.

On the other hand, the country's groundwater level is gradually declining as a result of continued groundwater abstraction for industrial use and crop cultivation^7^. However, in the globalization race, it is impossible to avoid industrialization. In this context, an effective method of repurposing industrial wastewater for agricultural irrigation is urgently needed to reduce wastewater pollution, groundwater dependency for agricultural usage, and wastewater treatment costs. After discovering that wastewater contained both water and substances that benefited the soil and increased agricultural yields, the idea of utilizing wastewater to irrigate was born around 3000 B.C.^8^. Commonly, reusing wastewater in agriculture is considered a deleterious practice since it may introduce pollutants to the environment, spread waterborne diseases, generate odor problems and result in aversion to the crops. Nevertheless, this kind of reuse resulted in some benefits for soils, crops and farmers and evident in the previous reports^9^. It has been revealed that the wastewater used for irrigation contains a significant load of biodegradable organic material (carbon and nitrogen) as well as most of the mineral macronutrients (e.g. phosphorous, potassium, magnesium and boron) and micronutrients (e.g. molybdenum, selenium and copper) which are necessary for the growth and yield of crops^10–12^.

Meanwhile, accumulating organic matter in soil by wastewater irrigation might be helpful because it can improve the physical structure of the soil, increase soil microbial activity, and improve soil performance as a pollution filter and degrading media^12,13^. Organic pollutants (e.g. polyaromatic hydrocarbons and polychlorinated biphenyls) and pathogenic microbiological organisms, on the other hand, account for a portion of the organic matter in wastewater^14,15^. As a result of continual pollution and pH changes, the capacity of soils to store toxic metals is diminished, and heavy metals such as cadmium (Cd), lead (Pb), chromium (Cr), and nickel (Ni) may be released into groundwater^16,17^. As a result, there is a detrimental influence on crop vegetation, health hazards, soil qualities, and the environment^18^. Besides, human is subjected to a direct impact on health with deleterious heavy metals by contamination of soils and transferred to them through the food chain of consuming contaminated crops^12,19–21^.

In some countries, the reuse of wastewater in agriculture is now recognized as a cost-effective environmental approach based on the benefits^22,23^. Large amounts of untreated wastewater have been utilized in agricultural irrigation for a long time in Mexico, China, India, and Pakistan, for example^24^. The World Health Organization estimates that untreated wastewater is used to irrigate over 20 million hectares around the world^25^. It's also been reported that in some cities, wastewater is used to irrigate up to 80% of the vegetables consumed locally^26^. Furthermore, the infiltration of wastewater into soil, particularly untreated wastewater, poses a substantial risk of pollution, not only to soil and crops, but also to surface and subsurface water sources surrounding the irrigated area^27,28^. Recently, concerns have risen regarding the potential risks of the pollutant’s loading to the soil and within crops and their continuous entry into each component of the environment through wastewater^13^. In this sense, several studies have been conducted to sort out the physiochemical properties of industrial (loom dye, tannery, textile etc.) wastewater, their effect on the growth, yield, nutrition qualities, heavy metals accumulation in soil, crops and health risk assessment^13,29,30^ using only the mixed effluents as the study materials. As a result of the harmful and aesthetic effects of textile effluents on receiving waters and irrigation, treatment of textile effluents is of interest. While substantial research has gone into developing effective treatment technologies for wastewaters containing azo dyes and other harmful compounds, no single solution has been shown to be adequate for dealing with the wide range of textile wastes^31^. The most popular and, so far, efficient approach is to treat dyeing industry wastewater with effluent treatment plants (ETPs). However, in most poor countries, such as Bangladesh, where many textile dyeing industries operate without an ETP, while the concept of CETP (common effluent treatment plant) is not working due to its high operational and maintenance costs. Furthermore, there are seventeen steps in knit fabric dyeing process where the wastewaters of all sections are not strongly polluted. In some dyeing process, both chemicals and fresh water are used and some other process only fresh water is used for fabric washing and cleaning after a chemical treatment. Haque^32^ stated that about 50% of wastewater generated from a dyeing process are polluted and needs to be treated and the rest of the wastewater can discharge directly or subjected to very mild treatment.

This feature creates the further scope to work on the specific stages of dyeing process focusing on the needs of the farmers who use wastewater for irrigation having essential nutrients, policy makers and industry owners to make proper legislation aimed at promoting textile wastewater utilization in agricultural irrigation after ETP with minimum cost. Meanwhile, in our preliminary studies on different stages of textile dyeing wastewater were evaluated their impact on the seed germination and growth of country bean and red amaranth^4,5^. But there were some issues are still need to be addressed through a comprehensive study to perform more accurate risk assessment studies on contamination of soil and crops using different stages of textile dyeing wastewater. Therefore, it has been hypothesized that different stages of textile dyeing wastewater might be varied as per the physiochemical traits and remarkably effect on the crop growth and soil pollution properties (Supplementary Fig. 1A,B). In the present study we have tried to optimize one or more stages of textile dyeing wastewater for irrigation emphasizing the physiochemical properties of the studied wastewater with their positive and negative impacts on the growth, yield and nutritional composition of tomato; how this practice can benefit soil properties through maximizing plant nutrient use efficiency and at the same time posing a risk of heavy metals contamination through pollution load index and transfer to the irrigated tomato plants.

## Methods

### Experiment design

The study was conducted with tomato (*Solanum lycopersicum* L.) as the test plant at the Horticultural Research Farm and the Soil Science Laboratory of the Bangabandhu Sheikh Mujibur Rahman Agricultural University, Salna, Gazipur, Bangladesh. To complement our study, the tomato plant was chosen as the most exploited fruiting vegetable and grown in diversified habitats of household garden, commercial field and the net house. Moreover, tomato, as a significant crop, has long fruiting duration of about 3 months with high nutrient and fertilizer requirements^33^. Hence, tomato was supposed to be the right plant of studying the textile dyeing wastewater (TDW) impact on the growth behavior, plant nutrient efficiency and pollution load index of the irrigated soil for 3 months duration that resembles most of the vegetables’ duration requirement.

The sources of irrigation water for tomato cultivation were the different stages of textile dyeing wastewater (Supplementary Fig. 1A) and ground water was used as control. It has been reported that in a dyeing process there are 17–20 different stages are involved and about 150 L fresh water is required for dyeing 1 kg of cotton goods^4,5^. We assumed that if we could identify some stages of TDW suitable for irrigation then this water could be discharged without ETP and will bear benefits for the farmers and industry owners to reduce the ETP cost. For example, 20ton capacity dyeing industry required total of 150 × 20,000 = 3,000,000 L water per day. For running each stage of a dyeing process, it requires water at the rate of 1:10 i.e., for 1 kg cloth 10times water and for 20,000 kg cloth it takes 2,00,000 L water for one stage. If we could save three stages then the amount of water that could be saved as 600,000 L. Ultimately, this would be helpful to save 20% of total water requirement/day.

Before irrigation the water samples and soil used for tomato cultivation were analyzed to sort out the physiochemical characteristics. No pesticides and fertilizers were applied during the crop production to avoid the inclusion of foreign nutrients or chemicals to the existing study samples. The plants were grown in the earthen pots and kept under a net house structure, covered with white polythene sheet from the top to avoid the mixture of rainfall water to the studied samples. Subsequently, growth, yield and biochemical parameters were analyzed at different days after planting till harvest. After harvest, the irrigated soils were further analyzed to figure out the plant nutrient use efficiency by the cultivated plant (tomato); total residue in the soil after harvest; heavy metal enrichment factor and pollution load index of the wastewater irrigated soils.

### Characterization of wastewater and groundwater

#### Selection of textile dyeing factory

Wastewater samples were collected from the Tex Euro BD Ltd, 20 tons dyeing capacity knit composite factory which is situated 10 km away from the study area. Due to the closer distance, it took very short time to collect the wastewater samples and analysis before changing physicochemical properties of wastewater.

#### Selection of different stages textile dyeing wastewater

There are several steps in knit dyeing process. Scouring and bleaching, dyeing, soaping, fixing and softening are very common steps in cotton (yarn and knit) fabric dyeing process. In some processes only less, harmful organic chemical is used. In enzyme treatment process, bio-polish enzyme is used. Enzyme is actually protein-based microorganism. Therefore, it has been assumed that the wastewater coming from this processing step would have no significant harmful impact on environment. On the other hand, in soaping, fixing and softening treatment process, organic based eco-friendly chemicals are used in most of the cases. In some process, only fresh hot or cold water is used to wash the fabric for cleaning. Therefore, effluents after different processing steps were selected for irrigation with the diversified features as treatment variables to cultivate tomato.

**Table:** 

Sample ID	Sample collection step	Sample ID	Sample collection step
T_1_	Groundwater (Control)	T_5_	Neutralization treatment
T_2_	Second wash after scouring and bleaching	T_6_	2nd wash after Soaping
T_3_	Enzyme treated water	T_7_	Fixing treatment water
T_4_	2nd wash after bath drain	T_8_	Mixed effluent from equalization tank before ETP

#### Sample collection and preservation for analyses

The effluents samples were carried out in 120 L plastic container for using as irrigation purpose of tomato cultivation and labeled as T2-T8 and ground water was also preserved with the marking of T1 (control). Samples for effluents characterization were taken in two liters plastic bottles with good stoppers from the plastic container. Two drops of concentrated HNO_3_ were added to 1 L of sample of each treatment for heavy metal analysis while the rest 1 L sample was kept without adding any acid for the analysis of major ions. Bottles were thoroughly washed with 1 M HCl and rinsed several times with de-ionized water before sample collection. Sampling was carried out using the grab method except mixed effluent^34^. Among the seven studied TDW, six different stages of effluents were collected from the processing steps of dyeing whereas mixed effluent sample was taken from equalization tank those were discharged before purifying at ETP. Groundwater (GW) sample was collected as control treatment from supplied deep tube well water near the experimental site that is commonly used for irrigation purpose in the research field. The samples were brought to the Environmental laboratory, Dhaka University of Engineering and Technology (DUET), Gazipur and preserved at 4 °C in a refrigerator to analysis the various physio-chemical characteristics adopting the procedures outlined in the standard procedures^35^.

#### Laboratory analysis of textile dyeing wastewater

The physicochemical parameters such as pH, temperature, color, electrical conductivity, dissolved oxygen (DO), chemical oxygen demand (COD), biochemical oxygen demand (BOD), total suspended solids (TSS), total dissolved solids (TDS) and nitrate (NO_3_^–^), phosphate (PO_4_^–3^), sulphate (SO_4_^–2^), chloride (Cl^–^) and seven heavy metals were selected for their estimation in the selected TDW, GW and soil based on the earlier reports and the chemicals used for dyeing process. The pH and temperature of wastewater was determined using portable HACH pH meter. The primary buffers of pH 4.0 (Phthalate), pH 7.0 (phosphate) and pH 10.0 (borax) were used as references to calibrate the pH meter. Determination of other parameters such as colour, nitrate (NO_3_^–^), sulphate (SO_4_^–2^), phosphate (PO_4_^–3^) were carried out in the laboratory using DR-2800™ portable spectrophotometer. Electrical conductivity was determined by conductivity meter (EC150, HACH). Biochemical oxygen demand (BOD) was measured by dilution method^35^. Keeping samples for 5 days in an incubator at 20 °C after measuring initial DO of samples, dissolve oxygen (DO) was measured by chemical method. Chemical oxygen demand (COD) was determined by dichromate digestion method. Chloride was determined by Mohr’s silver-nitrate method. Suspended solids (SS) were measured gravimetrically while total solid was obtained by the sum of SS and TDS. Heavy metals (iron, copper, zinc, lead, cadmium, nicle and chromium) determination was carried out using Atomic Absorption Spectrophotometer (SPECTRA A.A-55B, VARIAN, and Australia) as per standard methods.

### Characterization of soil

#### Soil sample collection

Vegetables growing soil was collected (0–30 cm depth) from the cultivation site in three distinct positions (as three replications) of the Horticulture research farm of BSMRAU, before the start of the experiment. Cow dung and compost were mixed with the experimental soil as required amount. This soil was poured into experimental plastic pots for cultivation. After harvesting of the cultivated crop, soil sample were also collected (0–30 cm depth) from every pot separately according to the treatments and kept into air tight polythene packet labeled with treatments to analysis the different parameters of soil after irrigation with TDW and GW. The samples were collected from the three distinct sites of each pot representing three replications of dataset.

#### Sample preparation and laboratory analysis

Soil samples were air-dried, grind and passed through a 2 mm size sieve. Then these samples were analyzed for evaluating the soil properties using standard methods.

**Table:** 

Parameters	Method	References
Soil pH	Glass Electrode pH Meter method with soil water ratio 1:2.5	^36^
Organic carbon	Wet Oxidation method	^37^
Phosphorus	Bray and Kurtz’ method	^38^
Nitrogen	Kjeldahl method	^39^
Sulphur	Turbid metrically as barium sulfate	^40^
Zinc	AB-DTPA method	^41^
Heavy metals (iron, copper, lead, cadmium, nickel, chromium)	AAS -digestion code 309	^42^

### Pot preparation for tomato cultivation

Tomato (*Solanum lycopersicum*) seeds were collected from the Horticulture department and sown in the nursery of the same department. The plants were maintained with following the scientific ethics to avoid genetic resources claim. The Tomato seedlings at 28 d old were grown in the earthen pots by following randomized complete block design (RCBD) with three replications. Seventy two (72) pots of 37 cm diameter and 50 cm height were filled with 10 kg of garden soil. Three pots of each treatment were assigned for growing 2 plants per plot and repeated for three times. Therefore, each experiment unit has 6 plants per treatment and triplicates samples were used for analyses to get average results. Fruits were harvested at the optimum maturity stage from all the treated pots and experiment continued up to 90 days, being enough to construe the maximum maturity which is known as days after transplanting (DAT) period. Tomato plant was irrigated with GW (control) and TDW (7 treatments) on an average at 6 six days interval of the total growing durations with 1 L of water in each irrigation schedule. Regarding the irrigation schedule we tried to reduce the leaching and waterlogged condition and used similar amount of water for all the treatment in each pot. However, frequency of irrigation was varied over the total duration of tomato cultivation for 90 days to avoid water deficit to plants. Within the first 4 weeks after planting irrigation was done 4 days interval; afterwards, it was done at 6 days intervals for 7 weeks during the vegetative and flowering-fruiting stage. Finally, irrigation was done at 8 days intervals for the last 2 weeks of the final stage of growing that revealed irrigation scheduled on an average at 6 days interval. Thus, 15 L of each irrigation water treatment was used for watering these pots. This amount of irrigation water was found enough to enhance the vegetative and reproductive growth of tomato without showing any moisture deficiency. This might be happened due to the low evapotranspiration under the polytunnel experimental condition. The plant samples (morphological and fruit) were collected from three replications at each plot for each water treatment for data collection and further analyses. The experiment was conducted under the shed house for controlling the addition of rain water during cultivation period.

### Growth and yield attributes of tomato

#### Plant height (cm)

Plant height was measured by measuring tape from the tip of the root to the tip of the leaves of the plants. Measurement was taken from 4 randomly selected plants of a treatment from each replication and average values were calculated.

#### No. of leaves per plant

Leaves per plant were counted from 4 plants of each replication for every treatment and average was calculated at that time.

#### Leaf length and width (cm)

Leaf length and width was measured randomly in cm on 5 leaves by measuring scale from each plant. This measurement was taken from 4 plants of each treatment of each replication and average was calculated at the same time.

#### Total no. of fruits

Total no. of fruits per cluster appeared after pollination and final harvested fruits from the respective cluster were counted from 4 plants of each treatment and average was calculated at that time. These data were used to calculate the fruit abortion (%) per treatment using the following formula-

1 $$Fruit\; abortion \left( \% \right) = \frac{{{\text{Fruits}}\;{\text{per}}\,{\text{cluster}}\;{\text{after}}\;{\text{pollination}} - {\text{Fruits}}\;{\text{after}}\;{\text{final}}\;{\text{harvest}}}}{{{\text{Fruits}}\;{\text{per}}\;{\text{cluster}}\;after\;pollination}} \times 100$$

#### Fruit length and width (mm)

Fruit length was measured randomly in mm on 5 fruits per treatment from each plant by digital slide calipers. Fruit diameter was measured from the previously selected same fruit samples in mm by digital slide calipers in the middle portion of the fruit. Measurement of randomly selected fruits was repeated 3 times from each treatment and average values were calculated.

#### Yield per plant (g)

Fruit yield per plant was calculated based on the individual fruit weight measured by the digital weighing scale. Individual fruit weight was taken from the selected 5 randomly selected fruits of 4 plants for each treatment and repeated for 3 times to make average.

### Biochemical analyses

#### Extract reparation for ascorbic acid estimation

Ten (10) gram of sample was weighed and taken in a warring blender (MX-7985, National, Malaysia). The sample was homogenized with warring blender by adding 50 ml distilled water. The homogenized solution was transferred into a 100 ml volumetric flask and then centrifuged for 20 min at the speed of 4,000 rpm. The supernatant liquid was collected in the 100 ml volumetric flask again. This was the extract solution for the determination of ascorbic acid.

#### Amount of ascorbic acid in fruit (mg/100 g)

The ascorbic acid was determined as per the procedure described by Pleshkov^43^. Ten ml of the extract was taken in a conical flask. Then 5 ml of 5% KI, 2 ml of 2% starch solution, 2 ml of 100% glacial acetic acid were added to it and shaken vigorously. Finally, it was titrated with 0.001 N of KIO3 solutions from a burette till the permanent pink color appeared. The volume of KIO3 solution required for the titration was noted from the burette reading and free ascorbic acid content was quantified using the following formula:

2 $${\text{Ascorbic}}\;{\text{acid}}\;{\text{content}}\;\left( {{\text{mg}}/{\text{l}}00{\text{ g}}} \right) \, = \frac{{\left( {FV1V2} \right)}}{{\left( {V3W} \right)}} \times 100$$

where F, 0.088 mg of Ascorbic Acid per ml of 0.001 N KIO_3_; V1, Titrated volume of KIO_3_ ml; V2, Total volume of the sample extract ml; V3, Volume of the extract (ml) taken for titration; W, Weight of the sample taken (g).

#### Estimation of β-carotene in fruit (mg/100 g)

One gram of sample was crushed thoroughly and mixed with 10 ml acetone: hexane (4:6) solution. This sample was centrifuged and optical density of the supernatant was measured by double beam spectrophotometer (Model: APEL, UV–VIS Spectrophotometer, PD—303 UV, PD 33-3-OMS-101 b, Japan) at 663 nm, 645 nm, 505 nm and 453 nm. Calculation was done by the following formula^44^.

3 $$\beta{\text{-Carotene }}\left( {{\text{mg}}/{\text{l}}00{\text{ g}}} \right) \, = 0.26\left( {{\mathbf{OD}}663} \right) + 0.452\left( {{\mathbf{OD}}453} \right) - 1.22\left( {{\mathbf{OD}}645} \right) - 304\left( {{\mathbf{OD}}505} \right)$$

where bold figure indicates optimal density.

#### Estimation of chlorophyll (mg/g)

Chlorophyll was estimated on fresh weight basis by scratching the peel of the samples with 80% acetone by using Double Beam Spectrophotometer (Model: APEL, UV–VIS Spectrophotometer, PD—303 UV, PD 33-3-OMS-101 b, Japan). Different chlorophylls were estimated using the following equations proposed by Witham et al.^45^. In brief, five mg peel of tomato was taken. The sample was dipped into 80% acetone in a test tube and the volume was made up to 25 ml. The test tube was covered with aluminum foil and the sample was kept over forty eight (48) hours in a dark place. Finally, the absorbance of the filtrate was taken by spectrophotometer at 663 nm and 645 nm respectively. The chlorophyll content of sample was calculated by the following formula:

4 $${\text{Chlorophyll}}\;{\text{a}}\;\left( {{\text{mg}}/{\text{g}}} \right) \, = \frac{{\left[ {12.7 \times \left( {OD663} \right) - 2.69\left( {OD645} \right)} \right] \times V}}{W \times 1000}$$

5 $${\text{Chlorophyll}}\;{\text{b}}\;\left( {{\text{mg}}/{\text{g}}} \right) = \frac{{\left[ {22.9 \times \left( {OD645} \right) - 4.68\left( {OD663} \right)} \right] \times V}}{W \times 1000}$$

6 $${\text{Chlorophyll}}\;{\text{total}}\;\left( {{\text{mg}}/{\text{g}}} \right)\; = \frac{{\left[ {20.2 \times \left( {OD645} \right) + 8.02\left( {OD663} \right)} \right] \times V}}{W \times 1000}$$

where OD_645_, Optical density at 645 nm wave length; OD_663_, Optical density at 663 nm wave length; V, Volume of the extract; W, Fresh weight in grams of the tissue extracted.

### Plant nutrient use efficiency (%)

Plant nutrient use efficiency (PNUE) depends on the plant’s ability to take up nutrients efficiently from the soil, but also depends on internal transport, storage nature, root growth, root architecture, irrigation management and remobilization of nutrients. In the present study it has been assumed that the essential plant nutrients available in the GW and TDW could be accumulated in the pot soil by the irrigation frequencies throughout the plant growing period. From these accumulated nutrients plant will uptake as per their requirement and might be expressed their growth and yield attributes and also bio-accumulated in different plant parts. The rest amount of nutrients might be left in the soil as residue. Therefore, PNUE was determined as per the procedure of Paul et al.^46^ with some modifications by the following formula:

7 $${\text{PNUE }}\left( {\text{\% }} \right) = \frac{{{\text{Nutrient}}\;{\text{Total}}\;{\text{Supply}} - {\text{Nutrient}}\;{\text{Residue}}\;{\text{in}}\;{\text{Soil}}}}{{{\text{Nutrient}}\;{\text{Total}}\;{\text{Supply}}}} \times 100$$

where

Nutrient _Total Supply_ = Total nutrient supply to the cultivated soil indicates the amount of nutrient supplied by 15 time irrigation frequencies with 1 L water of each irrigation + the amount of existing nutrient in the respective fresh soil before irrigation;

Nutrient _Residue in Soil_ = The residual amount of nutrient in the soil indicates the amount of nutrients remained in the cultivated soil that determined after subtracting the used amount of nutrient from the total supply of nutrient in the respective soil sample^46^.

### Enrichment factor (EF)

The term enrichment factor depicts the degree of contamination of soil in terms of accumulation of heavy metal in the soil through irrigation wastewater in compare to the heavy metal in the uncontaminated soil^47^. The enrichment factor (EF) for heavy metals accumulated in tomato cultivated soil irrigated with different stages of TDW and GW was estimated by the following equation-

8 $${\text{Enrichment}}\;{\text{Factor }}\left( {{\text{EF}}} \right) = \frac{{{\text{Mean}}\;{\text{content}}\;{\text{of}}\;{\text{individual}}\;{\text{metal}}\;{\text{in}}\;{\text{soil}}\;{\text{of}}\;{\text{the}}\;{\text{WW}}\;{\text{irrigated}}\;{\text{pots }}\left( {{\text{Sm}}} \right)}}{{{\text{Mean}}\;{\text{content}}\;{\text{of}}\;{\text{individual}}\;{\text{metal}}\;{\text{in}}\;{\text{soil}}\;{\text{of}}\;{\text{the}}\;{\text{GW}}\;{\text{irrigated}}\;{\text{pots }}\left( {{\text{Sc}}} \right)}}$$

where Sm = Total supply of metal in the soil through irrigation wastewater + existing metal content in the soil before irrigation;

Sc = Total supply of metal in the soil through irrigation with ground water (control) + existing metal content in the soil before irrigation.

### Pollution load index (PLI)

The degree of soil pollution load index for individual heavy metal was measured using the pollution load index (PLI) technique depending on soil metal concentrations exist for the specific duration of a crop. The following modified formula was used to measure the PLI in the tomato cultivated soils after crop harvest^48^.

9 $${\text{PLI}} = \frac{{{\text{Concentration}}\;{\text{of}}\;{\text{heavy}}\;{\text{metal}}\;{\text{in}}\;{\text{WW}}\;{\text{irrigated}}\;{\text{soil}}\;{\text{remained}}\;{\text{as}}\;{\text{residue}}\;{\text{after}}\;{\text{cultivation}}}}{{{\text{Concentration}}\;{\text{of}}\;{\text{heavy}}\;{\text{metal}}\;{\text{available}}\;{\text{in}}\;{\text{the}}\;{\text{GW}}\;{\text{irrigated}}\;{\text{soil}}\;{\text{after}}\;{\text{cultivation}}}}$$

### Sodium absorption ratio (SAR)

The sodium absorption ratio (SAR) is commonly used as an index for evaluating the sodium hazard associated with an irrigation water supply^49^. The SAR is defined as the square root of the ratio of the Na to calcium + magnesium (Ca + Mg). As different stages of TDW were used as irrigation treatment for tomato cultivation, therefore it is essential to sort out the sodium toxicity level of the TDW in compare with the GW for suitability justification of the TDW for vegetable cultivation. All cation measurements were expressed as milliequivalents per liter (meq/l). The SAR value was estimated as below-

10 $${\text{Sodium}}\;{\text{Absorption}}\;{\text{Ratio }}\left( {{\text{SAR}}} \right) = \frac{{{\text{Na}}^{ + } }}{{\sqrt {\frac{1}{2}\left( {{\text{Ca}}^{2 + } { } + {\text{ Mg}}^{2 + } } \right)} }}$$

### Statistical analyses

The data recorded were analyzed statistically and expressed in terms of mean values of the three replications of each variable and standard errors. Two-way analysis of variance (ANOVA) was done for hypothesis test at 1% level of significance. HSD Tukey mean comparison post hoc test was performed for mean comparison among the irrigation treatments in relation to the significant dependent variables. Moreover, correlation matrix, cluster analysis and principal component analysis (PCA) was done in taking account of the all the dependent variables to find out the most effective and significant contributing characters for TDW irrigation evaluation in tomato cultivation. All the analysis was done with the help of R-program (version 4.1.2) using “agricolae”, “ggplot2”, “corrplot”, “factominer”, “factoextra” packages.

## Results

### Physiochemical properties of irrigation water

#### Physiological properties

Physiological properties (pH, TDS, TSS, EC and color) of ground water and different stages of textile dyeing wastewater (TDW) were analyzed and compared with irrigation water quality standard guidelines (Table 1). These analyses show significant variations in their composition. Among the studied seven stages of TDW, pH was found in the range of 6.1 to 9.5 denoted acidic to alkaline properties. The pH of the five stages of TDW (T3, T5, T6, T7) and T1 (ground water) was recorded within the permissible limit (6.5–8) for irrigation water prescribed by^50^, while for T8, T2 and T4 it was exceeded that limit with the pH value of 9.5, 9.1 and 8.2 respectively. Accordingly, all the studied water sources for irrigation represented the TDS content within the irrigation standard limits with the value in the range of 300 to 1840, whereas T8 and T4 could be characterized as saline having the highest TDS of 3320 and 2070 than the guidelines. The EC indicates the total concentration of soluble salts in the sample. In this study, the EC value of the studied TDW samples varied from 480 to 900 that is at per with the T1 and within the safe limited of DOE^51^. However, T8 showed more than three times higher than safe limit (4200) followed by T4 (1350). Highly colored liquid effluents with pungent odor were observed in the T8, T4, T5, T7 and T6 samples in the chronological order of 1038 > 477 > 367 > 348 > 171 while the rest samples T1, T2 and T3 having the color estimation value of less than 100. With respect to DO, BOD and COD content, there was a significant difference observed among the studied water samples. The DO was found within the guideline limit (4.5–8) for all the studied samples except T8 (0.58) with the remarkable reduction than that of the others (Table 1). The BOD content exceeded the safe limit in the T8, T7 and T6 while the similar trend of results was also observed in case of COD with including two other stages of TDW T2 and T3 where COD was the highest compared to others.

**Table 1:** Physiochemical attributes of ground water and different stages of textile dyeing waste water.

Parameter^X^	Irrigation water^Y^								Guideline
	T1	T2	T3	T4	T5	T6	T7	T8	
**Physiological**									
pH	7.2 ± 0.1d	9.1 ± 0.1b	6.1 ± 0.15e	8.2 ± 0.2c	7.2 ± 0.1d	7.4 ± 0.1d	7.3 ± 0.2d	9.5 ± 0.1a	6.5–8^F^
TDS	300 ± 10 h	910 ± 10e	650 ± 15f	2070 ± 20b	1840 ± 20c	470 ± 5g	1290 ± 15d	3320 ± 20a	450–2000^F^
TSS	30 ± 2e	40 ± 3de	50 ± 5cd	60 ± 5bc	66 ± 4b	40 ± 1de	40 ± 2de	310 ± 10a	200^D^
EC (µs/cm)	350 ± 20g	850 ± 25cd	900 ± 20c	1350 ± 50b	550 ± 15ef	480 ± 10fg	700 ± 12de	4200 ± 200a	1200^D^
Color (point-co-unit)	16 ± 1h	97 ± 1f	67 ± 2g	477 ± 10b	367 ± 5c	171 ± 6e	348 ± 8d	1038 ± 13a	
**Nutrient**									
DO	6.5 ± 0.1a	5.85 ± 0.15b	6.12 ± 0.12b	4.58 ± 0.08d	5 ± 0c	5.80 ± 0.3b	4.77 ± 0.1cd	0.58 ± 0.08e	4.5–8^D^
BOD	1.5 ± 0.1f	68 ± 3d	2 ± 0f	23 ± 3e	83 ± 3d	143 ± 13c	203 ± 8b	223 ± 3a	100^D^
COD	4 ± 0.2h	755 ± 10a	610 ± 15b	98 ± 6g	195 ± 13f	317 ± 17e	393 ± 20d	450 ± 25c	400^D^
NO_3_^–^ N	1.5 ± 0.2a	0.8 ± 0b	0 ± 0d	0.6 ± 0.1b	0 ± 0d	0.3 ± 0c	0.8 ± 0.1b	0.8 ± 0.1b	2.2^D^
PO_4_^3–^ P	0.52 ± 0.02c	0.52 ± 0.02c	0.81 ± 0.1b	0.23 ± 0.03e	0.27 ± 0.02e	0.19 ± 0.04e	1.06 ± 0.06a	0.4 ± 0.1d	–
K	3.35 ± 4.9	2.35 ± 3.16	2.51 ± 2.94	1.75 ± 1.64	3.85 ± 6.2	4.46 ± 7.4	3.37 ± 4.01	0.94 ± 0.94	–
SO_4_^2–^ S	0 ± 0e	9 ± 1c	2.5 ± 0.5de	38 ± 3b	0 ± 0e	5 ± 1cde	8 ± 1cd	65 ± 5a	< 200^R^
Cl^–^	31 ± 1c	8 ± 0.5c	5 ± 0.2c	2500 ± 70b	58 ± 3c	64 ± 4c	42 ± 2c	2700 ± 100a	–
**Heavy metal**									
Cu	0.087 ± 0bc	0.077 ± 0c	0.091 ± 0b	0.089 ± 0b	0.004 ± 0d	0.005 ± 0d	0.006 ± 0d	0.11 ± 0.01a	0.2^F^
Zn	0.065 ± 0f	0.072 ± 0f	0.079 ± 0f	0.914 ± 0.01a	0.19 ± 0.01e	0.316 ± 0.02d	0.384 ± 0.01c	0.594 ± 0.01b	2.0^N^^,P^
Fe	0.025 ± 0g	0.028 ± 0f	0.032 ± 0e	0.087 ± 0c	0.004 ± 0h	0.033 ± 0.001d	0.341 ± 0b	0.731 ± 0a	5.0^N^^,P^
Pb	0.001 ± 0e	0.002 ± 0de	0.002 ± 0de	0.005 ± 0c	0.003 ± 0d	0.002 ± 0de	0.024 ± 0b	0.026 ± 0a	5.0^N^^,P^
Cd	0.001 ± 0h	0.005 ± 0f	0.002 ± 0g	0.098 ± 0b	0.067 ± 0d	0.006 ± 0e	0.088 ± 0c	0.103 ± 0a	0.01^N^^,P^
Ni	0.001 ± 0h	0.008 ± 0g	0.009 ± 0f	0.253 ± 0b	0.186 ± d	0.016 ± 0e	0.194 ± 0c	0.373 ± 0a	0.2^N^^,P^
Cr	0.001 ± 0h	0.002 ± 0g	0.004 ± 0f	0.254 ± 0b	0.126 ± 0d	0.089 ± 0e	0.134 ± 0c	0.329 ± 0a	0.1^N^^,P^

^X^All determinations were done in mg/L (ppm) if not other than specified. *TDS* total dissolved solids, TSS total suspended solids, *EC* electric conductivity, *DO* dissolved oxygen, *BOD* biological oxygen demand, *COD* chemical oxygen demand.

^Y^T1 = Ground water (control), T2 = 2nd wash after scouring and bleaching, T3 = Enzyme treated water, T4 = 2nd wash after bath drain, T5 = Neutralization treatment, T6 = 2nd wash after soaping, T7 = Fixing treatment water, T8 = Mixed effluent, Numeric values (±) are standard deviation and letters in the same row (a–h) are significant difference.

^Z^Not reported yet.

^D^DOE^51^.

^F^Ayers and Westcot^50^.

^N^NAS/NAE^52^.

^P^Pratt^53^.

#### Plant nutrient properties

The mean concentration of NO3-N, PO4-P, K and SO4-S those are the major essential plant nutrient were found in the TDW analyzed sample and revealed within the safe limit for irrigation water. However, the most concerning issue arose with the highest concentration of Cl- observed in the T8 and T4 TDW with the value of 2700 and 2500 mg/L respectively (Table 1).

#### Heavy metal properties

The concentration of heavy metals (HMs) such as Copper (Cu), Zinc (Zn), iron (Fe) and lead (Pb) was recorded within the safe agricultural irrigation limit in all the studied water samples (Table 1). Results depicted that the concentration of Cu, Fe and Pb was higher in T8 (0.11, 0.731 and 0.026 mg/L) and for Zn it was in T4 (0.914 mg/L). In case of other heavy metals, like cadmium (Cd), nickel (Ni) and chromium (Cr) were also exhibited the highest concentration in T8 (0.103, 0.373, and 0.329) followed by T4 < T7 < T5 and those crossed the agricultural irrigation standard. Among the analyzed TDW most of the heavy metal concentrations were revealed under safe limit in the order of T6 < T3 < T2.

### Fresh soil properties

Soil that commonly used for the vegetable cultivation was selected and analyzed. Different physiochemical properties along with heavy metal concentration was checked before wastewater being applied as irrigation treatment in tomato cultivation. Physiochemical properties confirmed that physical traits, plant nutrient content and heavy metals of the fresh soil were within the safe limit (Table 2).

**Table 2:** Physiochemical attributes of fresh soil.

Characteristics		Fresh soil^x^	Guideline
Physical	Texture	Sandy loam	–^Y^
	Cation Exchange Capacity (CEC)	2.95 (meq/100 g)	–
	Electric conductivity (EC)	0.74 (ds/m)	–
	pH	6.85	–
	Total dissolved solid (TDS)	689.59	–
Plant nutrient	Calcium (Ca + +)	25.12 (meq/100 g)	–
	Magnesium (Mg + +)	12.23 (meq/100 g)	-
	Carbonate (CO3–)	14.27 (meq/100 g)	–
	Bicarbonate (HCO3–)	173 (meq/100 g)	–
	Nitrate–N	294	–
	Phosphate-P	987	–
	Potassium-K	5.7	–
	Sulphate-S	5.51	–
	Chloride (Cl-)	21.8	–
Heavy metal	Cu	24.58	100^E^
	Zn	53.81	300^E^
	Pb	0.001	100^E^
	Cd	1.12	3.0^E^
	Ni	2.86	50^E^
	Cr	1.36	100^E^

^X^All determinations were done in mg/kg (ppm) if not other than specified,

^Y^Not reported yet.

^E^Ewers et al.^54^.

### Plant morphology as influenced by waste water irrigation

#### Plant height (cm)

**Figure 1:**
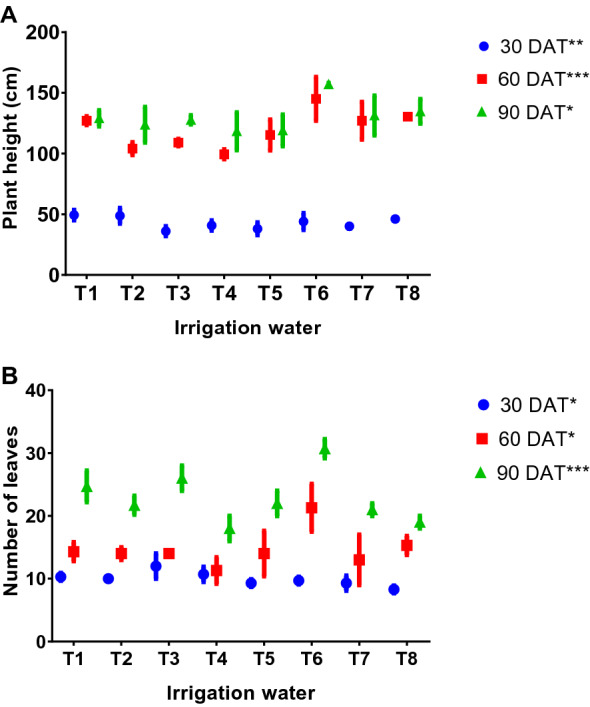
Plant height and leaves number of tomato as influenced by ground and different stages of textile dyeing waste water. (T1 = Ground water/control, T2 = 2nd wash after scouring and bleaching, T3 = Enzyme treated water, T4 = 2nd wash after bath drain, T5 = Neutralization treatment, T6 = 2nd wash after soaping, T7 = Fixing treatment water, T8 = Mixed effluent, DAT = Days after transplanting, * =  ≤ *P*0.05, ** = *P* ≤ 0.01, *** = *P* ≤ 0.001).

The effect of different stages of TDW irrigation on plant growth characteristics was evaluated at 30, 60 and 90 days after transplanting (DAT) with respect to plant height and leaves number that is displayed in Fig. 1A,B. As we observed the plant height was not significantly varied according to the treatments up to 30 DAT (Fig. 1A) while it was fluctuated at 60 DAT. Afterwards, there was a tendency to reduce the plant height at 90 DAT and found very close to the plant height of 60 DAT for the most of the treatments with the exception of T6 where the longest plant (157.0 cm) was produced. Meanwhile, the shortest plant was noticed in T4 (118.3 cm) at the end of 90 DAT.

#### Number of leaves

The number of leaves per plant was noticed as almost same up to 30 and 60 DAT for T2, T3, T4, T7, T1 whereas fluctuation was observed among the rest of the treatments T5, T6 and T8 (Fig. 1B). However, T6 provided completely remarkable findings than other where leaves number increased continuously and produced the maximum (30.7) at 90 DAT. On the other hand, leaves number were minimum in T4 (18.0) followed by T8 (19.0) at 90 DAT.

### Yield and yield attributes as influenced by waste water irrigation

#### Fruit abortion (%)

TDW significantly causes the fruit abortion and indicated that the highest fruit abortion was recorded in T8 (86.1%) which was statistically similar with T4 (84.2%) (Table 3). The lowest was found in the ground water treatment (53.6%) which was identical with T6 (59.6%).

**Table 3:** Yield and yield attributes of tomato as influenced by ground and waste water.

Irrigation water^Y^	Fruit abortion (%)	Fruit/plant	Fruit length (cm)	Fruit width (cm)	Yield/plant (g)
T1	53.6 ± 1.3d	34.0 ± 2.0a	35.6 ± 1.5a	33.5 ± 0.8ab	520.5 ± 67.2ab
T2	67.2 ± 1.6bc	20.7 ± 2.3cd	30.3 ± 1.1b	30.9 ± 2.2abc	324.7 ± 110.3bc
T3	74.4 ± 2.7ab	25.3 ± 2.3bc	32.7 ± 1.0ab	30.4 ± 1.2bcd	389.7 ± 98.4abc
T4	84.2 ± 4.7a	16.3 ± 1.5d	24.1 ± 1.5c	27.1 ± 0.5d	235.2 ± 37.9c
T5	76.4 ± 4.2ab	24.0 ± 3.5c	30.1 ± 0.7b	29.2 ± 1.2cd	378.6 ± 118.0abc
T6	59.6 ± 6.3cd	30.7 ± 2.5ab	35.0 ± 1.6a	34.2 ± 1.3a	565.6 ± 20.3a
T7	73.8 ± 3.8ab	20.0 ± 1.0cd	31.2 ± 1.0b	31.1 ± 0.4abc	275.9 ± 49.4c
T8	86.1 ± 5.9a	15.3 ± 1.2d	21.8 ± 1.4c	27.9 ± 1.1cd	197.8 ± 9.1c

^Y^T1 = Ground water (control), T2 = 2nd wash after scouring and bleaching, T3 = Enzyme treated water, T4 = 2nd wash after bath drain, T5 = Neutralization treatment, T6 = 2nd wash after soaping, T7 = Fixing treatment water, T8 = Mixed effluent, Numeric values (±) are standard deviation and letters in the same row (a–d) are significant difference.

#### Fruit per plant

The highest number of fruits per plant was produced in the ground water treated plant (34.0) followed by T6 (30.7) while remarkable reduction occurred in the T8 treated plant (15.3) followed by T4 (16.3) (Table 3).

#### Fruit length and width (cm)

Fruit characteristics regarding the length and width also represented the similar trend of previous phenomena where T8 (21.8 and 27.9 cm) and T4 (24.1 and 27.1 cm) treated plant produced the comparatively smaller size fruit (Table 3). Meanwhile, ground water treated plant (35.6 and 33.5) and T6 treated plant (35.0 and 34.2) were produced identically the largest fruit.

#### Fruit yield per plant (g)

The results on fruits number and size were profoundly contributed on the production of maximum yield per plant and it was evident in T6 (565.6 g) that was identical to T1 (520.5 g) (Table 3). As observed on another side, T8 and T4 treated plants produced the lowest yield per plant (197.8 g) followed by T4 (235.2 g) and T7 (275.9 g).

### Physiochemical attributes as influenced waste water irrigation

#### Chlorophyll a and chlorophyll b (mg/100 g)

The variation in the chlorophyll contents in the TDW generated tomato fruit was remarkable with respect to control (Table 4). The Chlorophylls (a, b) contents appeared to be significantly decreased at the T8 (0.12 mg/100 g; 0.06 mg/100 g) that was statistically identical with the findings of T4 (0.17 mg/100 g; 0.09 mg/100 g) a (0.83 mg/100 g). Meanwhile, the chlorophylls (a, b) content had dramatic increase in T6 treated tomato (0.83 mg/100 g; 0.34 mg/100 g) that was very close to control (0.76 mg/100 g; 0.28 mg/100 g).

**Table 4:** Physiochemical attributes of tomato as influenced by ground and waste water.

Irrigation water^Y^	Chlorophyll a (mg/100 g)	Chlorophyll b (mg/100 g)	β carotene (mg/100 g)	Ascorbic acid (mg/100 g)
T1	0.76 ± 0.04a	0.28 ± 0.03b	0.020 ± 0.002c	9.57 ± 0.33b
T2	0.46 ± 0.02b	0.18 ± 0.02c	0.011 ± 0.002d	7.65 ± 0.22d
T3	0.36 ± 0.03c	0.19 ± 0.02c	0.008 ± 0.001d	7.56 ± 0.11d
T4	0.17 ± 0.01de	0.09 ± 0.02d	0.001 ± 0.001e	6.23 ± 0.36e
T5	0.17 ± 0.01d	0.10 ± 0.01d	0.006 ± 0.002de	8.63 ± 0.21c
T6	0.83 ± 0.02a	0.34 ± 0.03a	0.038 ± 0.003a	11.04 ± 0.03a
T7	0.43 ± 0.03c	0.20 ± 0.01c	0.029 ± 0.004b	7.36 ± 0.11d
T8	0.12 ± 0.02e	0.06 ± 0.01d	0.001 ± 0.001e	5.07 ± 0.14f

^Y^T1 = Ground water (control), T2 = 2nd wash after scouring and bleaching, T3 = Enzyme treated water, T4 = 2nd wash after bath drain, T5 = Neutralization treatment, T6 = 2nd wash after soaping, T7 = Fixing treatment water, T8 = Mixed effluent, Numeric values (±) are standard deviation and letters in the same row (a–f) are significant difference.

#### β carotene and ascorbic acid (mg/100 g)

The lowest amount of β-carotene and ascorbic acid was determined in T8 treated fruit (0.001 and 5.07 mg/100 g) followed by T4 (0.001 mg/100 g; 6.23 mg/100 g). Nevertheless, β-carotene (0.038 mg/100 g) and ascorbic acid (11.04 mg/100 g) were the highest in T6 treated tomato fruits even in comparison with the control treatment (Table 4).

### Total supply and utilization of plant nutrients

It is well established that N, P, K, S are the essential plant nutrients having significant role in the growth, yield and physiochemical attributes. In the present study, total amount and percent use of these nutrients supplied by the ground water and different stages of TDW irrigation to the tomato cultivated soil were determined for better understanding their effectiveness on the plant growth (Table 5). Although ground water (T1) irrigated plant received the highest N (2963 mg), but use efficiency was the lowest (99.5%) while the highest N use efficiency (99.7%) was noticed by the T6 treated plants. In case of P and K, both the supply (9876 and 123.9 mg) and use efficiency (98.1 and 88.8, respectively) were recorded also the highest in the T6 TDW. Conversely, the lowest use efficiency of P and K (97.5 and 43.8%) was in the T8. Although T8 treatment provided the highest S (1030.1 mg) supply but use efficiency was the lowest (44.8) followed by T4 (42.2).

**Table 5:** Total supply and utilization pattern of plant nutrients (NPKS).

Plant nutrient	Irrigation water^Y^							
	T1	T2	T3	T4	T5	T6	T7	T8
**N**								
Supply (mg)	2963 ± 23a	2952 ± 20b	2940 ± 20d	2949 ± 22b	2940 ± 20d	2945 ± 20c	2952 ± 22b	2952 ± 19b
% Use	99.5 ± 0.02c	99.6 ± 0.04b	99.8 ± 0.02a	99.7 ± 0.02a	99.7 ± 0.04a	99.7 ± 0.02a	99.6 ± 0.02b	99.6 ± 0.04b
**P**								
Supply (mg)	9881 ± 25c	9881 ± 26c	9886 ± 24b	9877 ± 25e	9878 ± 25e	9876 ± 25e	9889 ± 24a	9879 ± 24d
% Use	97.3 ± 0.01h	97.4 ± 0.01g	97.5 ± 0.01f	97.9 ± 0c	98.0 ± 0b	98.1 ± 0a	97.8 ± 0d	97.5 ± e
**K**								
Supply (mg)	107.2 ± 73a	92.2 ± 47a	94.6 ± 44a	83.3 ± 40a	114.7 ± 93a	123.9 ± 111a	107.6 ± 60a	71.1 ± 14a
% Use	79.6 ± 10.1bc	82.4 ± 7.1bc	85.7 ± 5.3ab	83.7 ± 6.2abc	80.1 ± 11bc	88.8 ± 6.7a	79.0 ± 8.9c	43.8 ± 9.9d
**S**								
Supply (mg)	55.1 ± 0.03e	190.1 ± 15c	92.6 ± 7.5de	625.1 ± 45b	55.1 ± 0e	130.1 ± 8.7cde	175.1 ± 15cd	1030.1 ± 75a
% Use	90.2 ± 0a	80.9 ± 1.5b	82.5 ± 1.4b	42.2 ± 4.2c	82.2 ± 0b	83.2 ± 0b	89.6 ± 0.9a	44.8 ± 4.0c

^Y^T1 = Ground water (control), T2 = 2nd wash after scouring and bleaching, T3 = Enzyme treated water, T4 = 2nd wash after bath drain, T5 = Neutralization treatment, T6 = 2nd wash after soaping, T7 = Fixing treatment water, T8 = Mixed effluent, Numeric values (±) are standard deviation and letters in the same row (a–h) are significant difference.

### Enrichment factor (EF) and pollution load index (PLI)

The estimation of the enrichment factor (EF) of the heavy metals (HMs) and subsequent pollution load index (PLI) of the TDW irrigated soil is the prerequisite for explaining the health risk assessment of HMs through the transfer from the contaminated soil to the cultivated plants. In this study, EF and PLI was estimated keeping in mind this feature along with the aforementioned findings on the HMs in the TDW irrigated soil for tomato cultivation up to 90 days (Table 6). Results indicated that EF was the highest in T8 irrigated soil for Fe, Cu, Pb, Cd, Ni and Cr except Zn. Whereas, EF for Zn was the highest in T4 treatment. Moreover, in context with the PLI, T8 irrigated soil had the highest pollution load index (PLI) for Fe, Cu, Zn out of seven HMs and the lowest PLI for Cd and Ni. On the other hand, the highest PLI for Pb was observed in T7, for Cd it was in T5 and for Cr it was in T4 irrigated soil. Among the studied TDW, T6, T2 and T3 were not found as effective in increasing the EF and PLI of the irrigated soil as compared with the other stages of TDW. Moreover, the EF and PLI values of the T6, T3 and T2 were more or less similar with those of the control for most of the studied HMs.

**Table 6:** Enrichment factor (EF) by supplied irrigation water and Pollution load index (PLI) in the fresh soil after tomato cultivation.

Heavy metal	Irrigation water^Y^							
	T1	T2	T3	T4	T5	T6	T7	T8
**Fe**								
EF	1.000 ± 0.03g	1.001 ± 0.03f	1.003 ± 0.03e	1.022 ± 0.03c	0.929 ± 0.03h	1.003 ± 0.03d	1.117 ± d	1.262 ± 0.03a
PLI	1.0 ± 0g	1.5 ± 0f	1.0 ± g	286.0 ± 0b	20.5 ± 0e	29.5 ± 0d	214.0 ± 0c	362.0 ± 0a
**Cu**								
EF	1.019 ± 0.02bc	1.018 ± 0.02c	1.019 ± 0.02b	1.019 ± 0.02b	1.010 ± 0.02d	1.010 ± 0.02d	1.010 ± 0.02d	1.020 ± 0.02a
PLI	1.21 ± 0.2ab	1.26 ± 0.3ab	0.82 ± 0.1c	0.98 ± 0.1bc	1.03 ± 0.1bc	0.86 ± 0.1c	1.31 ± 0.1a	1.39 ± 0.1a
**Zn**								
EF	1.000 ± 0f	1.000 ± 0f	1.000 ± 0f	1.024 ± 0a	1.004 ± 0e	1.007 ± 0d	1.009 ± 0c	1.015 ± 0b
PLI	1.0 ± 0.1a	0.6 ± 0.1d	0.7 ± 0.1c	1.0 ± 0.1ab	0.6 ± 0d	0.7 ± 0.1c	0.9 ± 0.1b	0.95 ± 0.1ab
**Pb**								
EF	1.00 ± 0e	1.60 ± 0de	1.60 ± 0de	3.40 ± 0c	2.02 ± 0.12d	1.77 ± 0.05de	14.62 ± 0.42b	16.12 ± 0.42a
PLI	1.0 ± 0h	1.6 ± 0f	1.58 ± 0g	3.0 ± 0c	1.88 ± 0d	1.75 ± 0e	9.67 ± 0a	8.46 ± 0b
**Cd**								
EF	1.000 ± 0h	1.005 ± 0f	1.001 ± 0g	1.130 ± 0b	1.088 ± 0d	1.007 ± 0e	1.116 ± 0c	1.136 ± 0a
PLI	1.00 ± 0d	0.991 ± 0f	1.01 ± 0c	0.96 ± 0g	1.06 ± 0a	0.994 ± 0e	1.02 ± 0b	0.83 ± 0h
**Ni**								
EF	1.000 ± 0h	1.003 ± 0g	1.004 ± 0f	1.132 ± b	1.097 ± 0d	1.008 ± 0e	1.102 ± 0c	1.195 ± 0a
PLI	1.00 ± 0a	0.93 ± 0d	0.92 ± 0e	0.81 ± 0g	0.94 ± 0c	0.97 ± 0b	0.82 ± 0f	0.72 ± 0h
**Cr**								
EF	1.000 ± 0h	1.001 ± 0g	1.003 ± 0f	1.279 ± 0b	1.138 ± 0d	1.097 ± 0e	1.147 ± 0c	1.361 ± 0a
PLI	1.00 ± 0e	0.97 ± 0g	0.96 ± 0h	1.07 ± 0a	1.04 ± 0d	1.05 ± 0c	0.99 ± 0f	1.06 ± b

^Y^T1 = Ground water (control), T2 = 2nd wash after scouring and bleaching, T3 = Enzyme treated water, T4 = 2nd wash after bath drain, T5 = Neutralization treatment, T6 = 2nd wash after soaping, T7 = Fixing treatment water, T8 = Mixed effluent, Numeric values (±) are standard deviation and letters in the same row (a–h) are significant difference.

### Sodium absorption ratio (SAR)

For further exploration of the irrigated soil qualities and its consequence on plant growth issues, SAR from the soil residue was measured after irrigation with TDW and compared with the ground water (Table 7). Results indicated that concentration of Na + and Ca2 + were the highest in T8 treated soil followed by T4 and T7 accordingly. Inversely, T8, T4 and T7 irrigated soil showed the 1st, 2nd and 3rd lowest Mg2 + content in sequence. These ultimately increased the SAR values of T8, T4 and T7 treated soil with the value of 21.46, 10.10 and 4.60 respectively and turned the fresh soil into “severe” or “slight to moderate” categorized saline soil. The rest of the TDW treated soil specifically T2, T3 and T6 did not transformed soil to salinity in that level in correspond with the control.

**Table 7:** Classification of Irrigation water based on Sodium Absorption ratio (SAR).

Irrigation water^Y^	Concentration in (meq/L)			SAR	Degree of restriction^F^ (< 3.0 = None, 3.0–9.0 = Slight to moderate, > 9.0 = Severe)
	Na^+^	Ca^2+^	Mg^2+^		
T1	18.03 ± 0.15f	9.53 ± 0.31g	419.3 ± 1.53a	0.87 ± 0.00g	None
T2	20.67 ± 1.53f	18.1 ± 0.36f	396.0 ± 2.0b	1.02 ± 0.08fg	None
T3	26.17 ± 0.76e	12.3 ± 0.64g	375.0 ± 2.65c	1.33 ± 0.03ef	None
T4	189.57 ± 1.25b	65.0 ± 1.0b	287.7 ± 3.21f	10.10 ± 0.13b	Severe
T5	49.67 ± 2.08d	29.7 ± 2.08d	312.0 ± 2.0e	2.69 ± 0.10d	None
T6	27.67 ± 1.53e	24.0 ± 2.0e	354.0 ± 3.0d	1.42 ± 0.08e	None
T7	87.00 ± 1.0c	52.7 ± 2.08c	305.0 ± 4.58e	4.60 ± 0.09c	Slight to moderate
T8	391.33 ± 2.52a	116.0 ± 2a	216.7 ± 2.52g	21.46 ± 0.24a	Severe

^Y^T1 = Ground water (control), T2 = 2nd wash after scouring and bleaching, T3 = Enzyme treated water, T4 = 2nd wash after bath drain, T5 = Neutralization treatment, T6 = 2nd wash after soaping, T7 = Fixing treatment water, T8 = Mixed effluent, Numeric values (±) are standard deviation and letters in the same row (a–g) are significant difference.

^F^Ayers and Westcot^55^.

### Correlation matrix and cluster analysis

**Figure 2:**
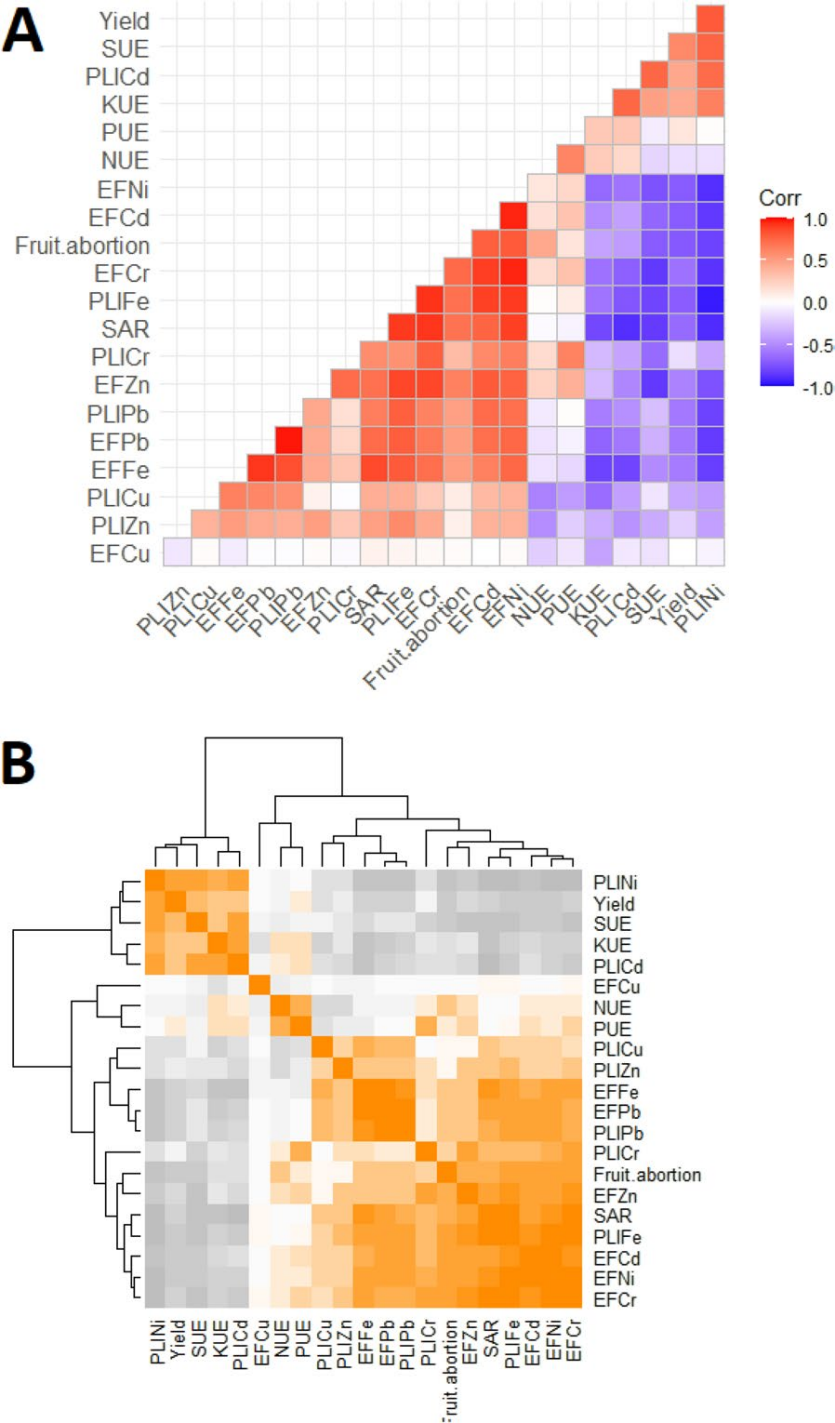
Correlation matrix in between and among yield attributes, nutrient efficiency, SAR, EF and PLI during and after tomato cultivation. [UE = use efficiency, EF = enrichment factor, PLI = pollution load index].

Correlation matrix between the variables showed moderate to strong positive and negative relationships with one or more variables (Fig. 2A). As observed, enrichment factor (EF) and pollution load index (PLI) of all the studied HMs with the exception of Cd and Ni were positively correlated with fruit abortion and SAR and negative correlation observed with tomato yield. Moreover, NPKS use efficiency (UE) were negative with the fruit abortion and SAR, while those were positive with yield. Based on the correlation, there were three main clusters were formed with the distinct deviations from each other (Fig. 2B). Cluster-I includes the variables of PLINi, Yield, SUE, KUE, PLICd; cluster-II grouped with EFCu, NUE, PUE and the cluster-III includes the rest of the total variables of 21 as indicated: PLICu, PLIZn, EFFe, EFPb, PLIPb, PLICr, Fruit abortion, EFZn, SAR, PLIFe, EFCd, EFNi and EFCr. From these cluster analyses it has been revealed that plant nutrients, yield variables are clearly distinguished than that of the heavy metals’ enrichment factor and pollution load index variables (Fig. 2B).

### PCA of TDW irrigated soil data

**Figure 3:**
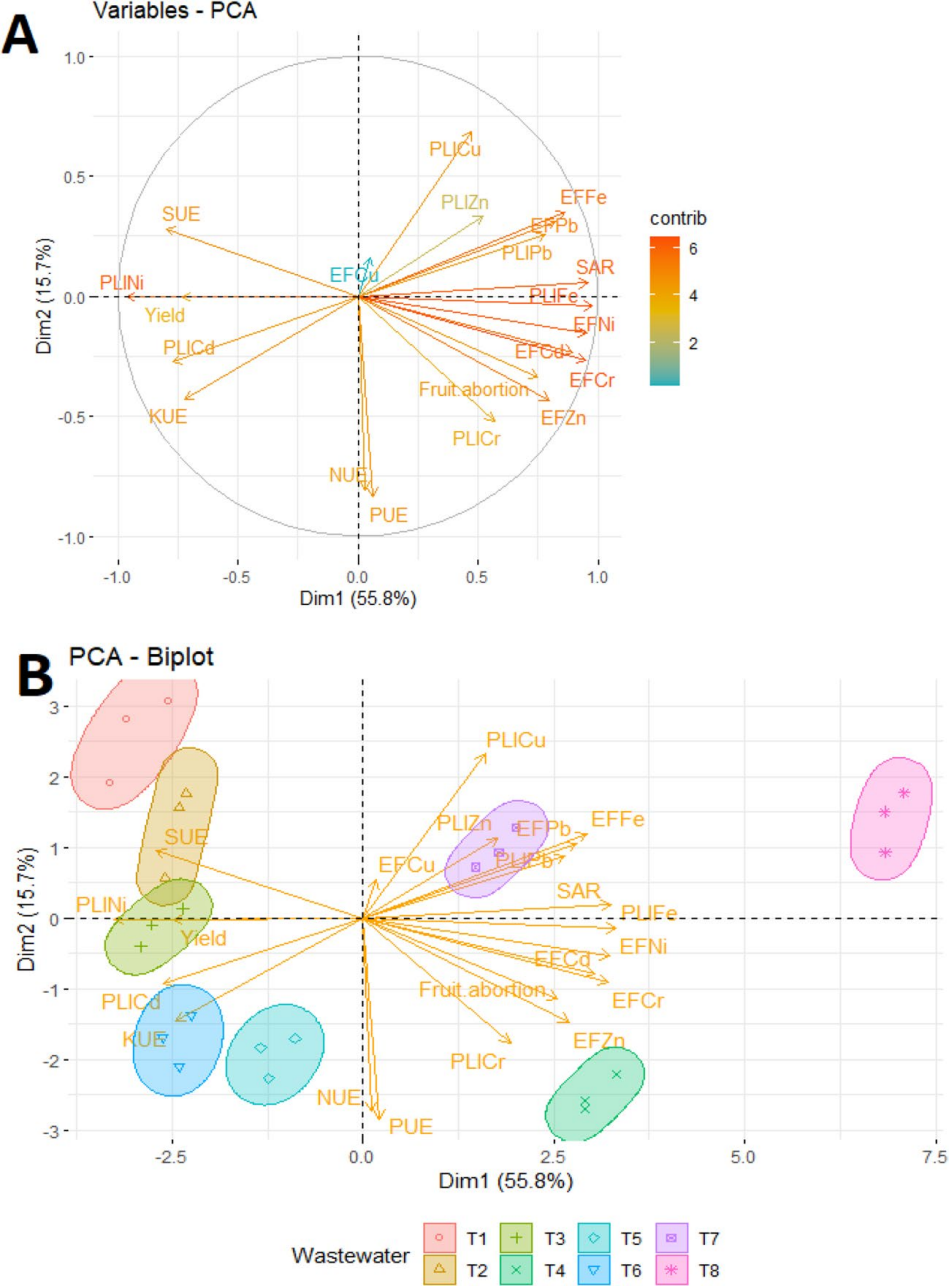
Principal Component Analysis (PCA) of yield attributes, nutrient efficiency, SAR, enrichment factor (EF) and Pollution load index (PLI) during and after tomato cultivation. [(**A**) PCA of the variables showing their major contribution, (**B**) PCA-Biplot analysis representing the clustering of ground water and different stages of textile dyeing waste water towards major contribution. [UE = use efficiency, SAR = sodium absorption ratio, T1 = Ground water (control), T2 = 2nd wash after scouring and bleaching, T3 = Enzyme treated water, T4 = 2nd wash after bath drain, T5 = Neutralization treatment, T6 = 2nd wash after soaping, T7 = Fixing treatment water, T8 = Mixed effluent].

Principal component analysis (PCA) employed to depict the relationship and impact of TDW wastewater irrigation on soil plant nutrient efficiency (PNUE); degree of pollution with heavy metals (HMs) by pollution load index (PLI) and tomato yield cultivated on those soils with precise evaluation of the large studied variables. PCA allowed us to deduce how certain variables were interconnected with each other to categories the TDWs after generating two significant PCs (Dim1 and Dim2) with total explained variance of 71.5% using the 21 variables (Fig. 3A,B). From the variables-PCA plot, it has been seen that the first PC1 (Dim1) explained 55.8% of total variables with positive loading on the PLIcu, PLIZn, EFFe, EFPb, PLIPb, SAR, EFNi, EFCd, EFCr, Fruit abortion, EFZn and PLICr. Meanwhile, 15.7% of the total variability was explained by PC2 (Dim2) with the positive loading on SUE (Fig. 3A).

In PCA-Biplot consists of PC1 vs PC2 (here Dim1 vs Dim2) drawn as vectors showed that seven stages of TDW and GW occupy different regions of the plots with well-defined patterns (Fig. 3B). Biplot simultaneously represents both the observations and the variables with a specific direction along with the specific PC axis. The direction of the variable arrows indicates the path in which contribution of the corresponding variable increases most, and the length of the arrows equals the rate of change in that direction. The clustering of irrigation water showed in ellipse indicated the significant impact of TDW irrigation on the yield; fruit abortion of tomato with negative correlation while grown in the heavy metals enriched and polluted soil. Among the TDW stages T8, T4, T7 distinctly fell into the opposite direction to the T2, T3, T5 and T6 with respect of the PC1. Considering the PC2, soils from the T8 and T4 irrigated pot were grouped in opposite side with the highest concentrations of Cu, Zn, Fe, Pb in T8 and Cd, Ni, Cr in T4. Regarding the EF and PLI of HMs variables, most of the EF and PLI were closely grouped together and associated with the cluster of T8, T7 and T4. Besides, T2, T3, T5 and T6 were clustered with SUE, PLICd, KUE showed the weak contributions. Furthermore, soil samples from the GW (control) with the lowest content of heavy metals were grouped in the negative side of the PC1 and positive side of the PC2, revealed the reduce influence of the heavy metals on the GW irrigated soil.

## Discussion

### Nutrients and heavy metal properties of TDW

Since wastewater is produced constantly and is always available, it is possible to make this benefit worthy for irrigation after explaining how the reuse of wastewater can compensate plant nutrients withdrawal through the constant nutrient input into the irrigated soil. In this study the quantified macronutrients (N, P, K, S) in the TDW revealed within the acceptable limits for agricultural use (Table 1). Furthermore, nonessential micronutrient Cl- showed higher levels in T8 and T4 of TDW than those in the safe limit.

On the other hand, different stages of TDW contained heavy metals (Cu, Zn, Fe) those are considered as micronutrients for plant were within the safe limits for agricultural purposes while, the nonessential heavy metals Pb, Cd, Ni, Cr exceeded the acceptable limit in the order of T8 < T4 < T7 < T5 treatment whereas the rest of the TDW T2, T3, T6 exhibited the heavy metals within the safe limit. These findings were also supported by the report of^56^.

### Relationship between plant nutrient use efficiency and tomato yield

To investigate the relationship between plant nutrients in TDW irrigation water and tomato yield, we monitored irrigation amounts, growth, yield attributes of tomato and calculated total supply and use% of N, P, K, S with their corresponding concentration (Tables 3, 4, 5). The total inputs of N, P, K, S in the irrigation water treatment showed minimum differences as same soil was used for tomato cultivation without extra fertilizer application for 90 days growing duration (Table 5). This variation was only occurred due to the differences in the concentration of the respective macronutrients in the specific stage of TDW. However, the utilization pattern of K and S was reduced two times in T4 and T8 than those of the other TDW. Whereas, N and P were used effectively by the plants irrigated with all of the TDW compared to ground water (T1). These findings were concurrent with the several reports that postulated the positive effects of wastewater reuse for agriculture through the improvement of soil fertility, nutrient amendment and as a result improved crop production^57–59^. Phytotoxicity and fruit quality is one of the most important identifiers to make decision on whether the TDW could safely be reused for tomato irrigation. Tables 3, 4 and Fig. 1 showed growth, yield and physiochemical attributes of tomato were remarkably hampered in T8 followed by T4 while T6 exhibited the best outcome. These findings substantiated with the previous observations where the wastewater rich with macronutrients have been suggested to secure nutrient recycling for plants and food chains and enhance agricultural production^60–62^.

These poor exhibition by T8 and T4 might be due to the excess concentrations of physical and heavy metal components (Table 1). Lellis et al.^63^ stated that textile dyes significantly negotiate the water aesthetic quality with increased BOD and COD which impair photosynthesis and inhibit plant growth, enter the food chain, provide recalcitrance and bioaccumulation, and may promote toxicity, mutagenicity and carcinogenicity. Goel^64^ reported the higher EC as an indicator of excess salinity that restricts water potentiality of the soil and impact on crop physiology and yield. On the other hand, Joshi et al.^65^ revealed that the higher concentration of Cr seriously reduced leaf chlorophyll, root, and shoot length. Whereas, the high level of Cd reflected visible symptoms of injury in terms of chlorosis, growth inhibition, browning of root tips and finally death^66^. For these reasons, use efficiency of P, K, S plant nutrient components were lower in T8 followed by T4 resulted tomato yield reduction. Comparatively T6 impact on better growth, yield and quality attributes that is primarily due to higher supplier and user efficacy of N, P, K. The growth was governed by the involvement of N through protein metabolism, P in the defense mechanism and K in stomata opening and closing process^67^. The obtained results also support the suggestion of^68^ who advocated the reuse of wastewater in arid and semiarid region of the world and reported significantly higher yield of melon and tomato obtained due to irrigation with effluents.

### Heavy metals enrichment and pollution load index of TDW irrigated soil

Heavy metals (HMs) enrichment factor (EF) and pollution load index (PLI) is a parameter used for better understanding the transfer pattern of HMs from TDW irrigated soil to plant in comparison with the ground water irrigated soil. Enrichment factor (EF) expressed proportionately higher supplier of heavy metals to the plant, while pollution load index (PLI) indicated maximum contribution in soil pollution. Here, higher EF with lower PLI of each HM mentioned the higher solubility and bioavailability for the plant, while higher EF with higher PLI indicated the lower bioavailability to the plant and deposited in soil. Obtained results showed T8 supply of most of the heavy metals (Fe, Cu, Pb, Cd, Ni, Cr) except Zn that is supplied by T4 to tomato plant after applying 15-L WW during the experimental period (Table 6). On the other hand, PLI of Fe, Cu and Zn into the T8 irrigated soil were the highest indicated higher deposition of these HMs into the soil. Conversely, the lowest PLI of Cd and Ni were also noticed in T8 irrigated sample indicated higher solubility and bioavailability to the plant. Consequently, Cd was easily absorbed by plants^57,69^ and most moveable metals which moved within organism by active ion pumps that normally transport Ca2 + ^70^. Moreover, the highest PLI for Pb was measured in T7 (9.67) followed by T8 (8.46), while for Cd it was in T5 (1.06) revealed lower bioavailability of Pb than Cd^71–73^. PLI of Cr was found the highest in T4 followed by T8 that expressed their higher deposition into T4, T8 TDW irrigated soil. Hamilton et al.^57^, indicated that Cr is less soluble in water and retained in the soil, while Ni absorbs loosely and more soluble in the soil solution. Copaciu et al.^74^, exposed that Cr from textile dye provided oxidative stress which is another problem associated with recalcitrant character, offering a considerable damage to the growth and development of plants, especially to photosynthesis and CO2 assimilation. Thus, T8 treatment produced the lowest yield of tomato compared to other treatment in the present study.

Furthermore, the quality of the different stages of TDW in the study are being used for irrigation purposes could possibly lead to other health implication through the most common route of food chain (soil–plant–food). In this aspect, sodium absorption ratio (SAR) of the TDW was determined for evaluating the sodium hazard associated with the respective irrigation treatment. As per the present findings, the highest SAR was measured in T8 (21.46) followed by T4 (10.10) and T7 (4.60) those possessed in descending order of T8 < T4 < T7 in respect to the concentration of Na + and Ca2 + (Table 7). The previous reports suggested that irrigation water having high SAR levels could lead to build-up of high soil Na levels over time, which in turn can adversely affect soil structure, infiltration rate, poor seedling emergence, and poor aeration^75^. Their findings in agreement with the present observation where T8 and T4 having the highest SAR value was responsible for reduction in tomato growth and yield attributes. Meanwhile, T8 and T4 TDW were not suitable for surface irrigation as per the SAR values that decided based on the wastewater quality guidelines for agricultural use and belonged to the severe saline group^50,55^. However, T7 was classified as slightly unsuitable for irrigation due to cause slight to moderate level salinity, while the rest of the stages of TDW T2, T3, T5 and T6 were revealed as suitable for irrigation as none of those cause salinity to the irrigated soil. The SAR values for the samples of T8, T4 were high because of having the highest amount of TDS, EC and Cl- those were supposed to be related factors enhancing the sodium, calcium and total salt content.

In addition, correlation matrix and PCA was done by considering all the studied variables to sort out the most promising factors responsible for optimizing the TDW as irrigation purpose for tomato cultivation. As observed, correlation matrix clearly explained that plant nutrients use efficiency (UE) were positively correlated with the yield and negatively with fruit abortion and SAR. EF and PLI of heavy metals were responsible in yield reduction and increased SAR with some few exceptions observed in Cd and Ni as per the different stages of TDW. Principal component analysis (PCA) also depicted and clustered the same variables as major contributors of yield improvement with T1, T2, T3 and T6 TDW irrigation treatment. In other side, EFFe, EFPb, EFZn, EFCd, EFCr, EFNi and other PLI were recognized as the major contributors of fruit abortion and SAR with the irrigation treatment of T8, T4 and T7.

## Conclusion

Based on the physiochemical characteristics of the different stages of TDW, impact analyses on soil properties, tomato growth and yield, there were no adverse effects of irrigation with the T2, T3, T6 stages TDW compared with ground water (T1), those satisfied agricultural water quality criteria. Total nutrients supplied with less heavy metals EF and PLI was obtained with T2, T3, T6 TDW those were highly correlated with tomato yield and nutrition data, indicating that there was a significant relationship between tomato yield and plant nutrient supply. Meanwhile, other stages of TDW like T8 < T4 < T7 < T5 chronologically showed higher EF and PLI for heavy metals and consequently transformed soil into severe to slightly to moderate saline category with increased SAR in reverse order. Therefore, this study optimizes the textile wastewater of the second wash after scouring and bleaching (T2); enzyme treated water (T3); second wash after soaping (T6) as suitable for agricultural irrigation.

Nevertheless, this study was not investigated the heavy metals accumulation pattern in different plant parts irrigated with TDW, despite the potential concern of increasing trends of heavy metals in the irrigated soil. Therefore, further studies are needed to explore the heavy metal accumulation and health risk assessment including more crops irrigated with different stages of textile dyeing wastewater (TDW).

### Declaration on plant handling with the relevant guidelines and regulations

Tomato (*Solanum lycopersicum*) was used as plant material in the present study. It has been declared that the tomato seeds were collected from the department of Horticulture, Bangabandhu Sheikh Mujibur Rahman Agricultural University, Gazipur-1706, Bangladesh. The tomato seeds are producing and preserving in the Horticulture department for using in the research purposes. The field study was conducted in the nursery of the same department with following standard procedures of cultivation and without violating the relevant guidelines and regulations of the plant handling process.

## Supplementary Information

Supplementary Information.
