# Supplementary material for: Optimizing textile dyeing wastewater for tomato irrigation through physiochemical, plant nutrient uses and pollution load index of irrigated soil
Source: Sci Rep. 2022 Jun 16;12:10088. doi: 10.1038/s41598-022-11558-1 (PMC9203507; doi:10.1038/s41598-022-11558-1)
Supplement: Supplementary file 1 — Supplementary Information. [file 41598_2022_11558_MOESM1_ESM.docx]

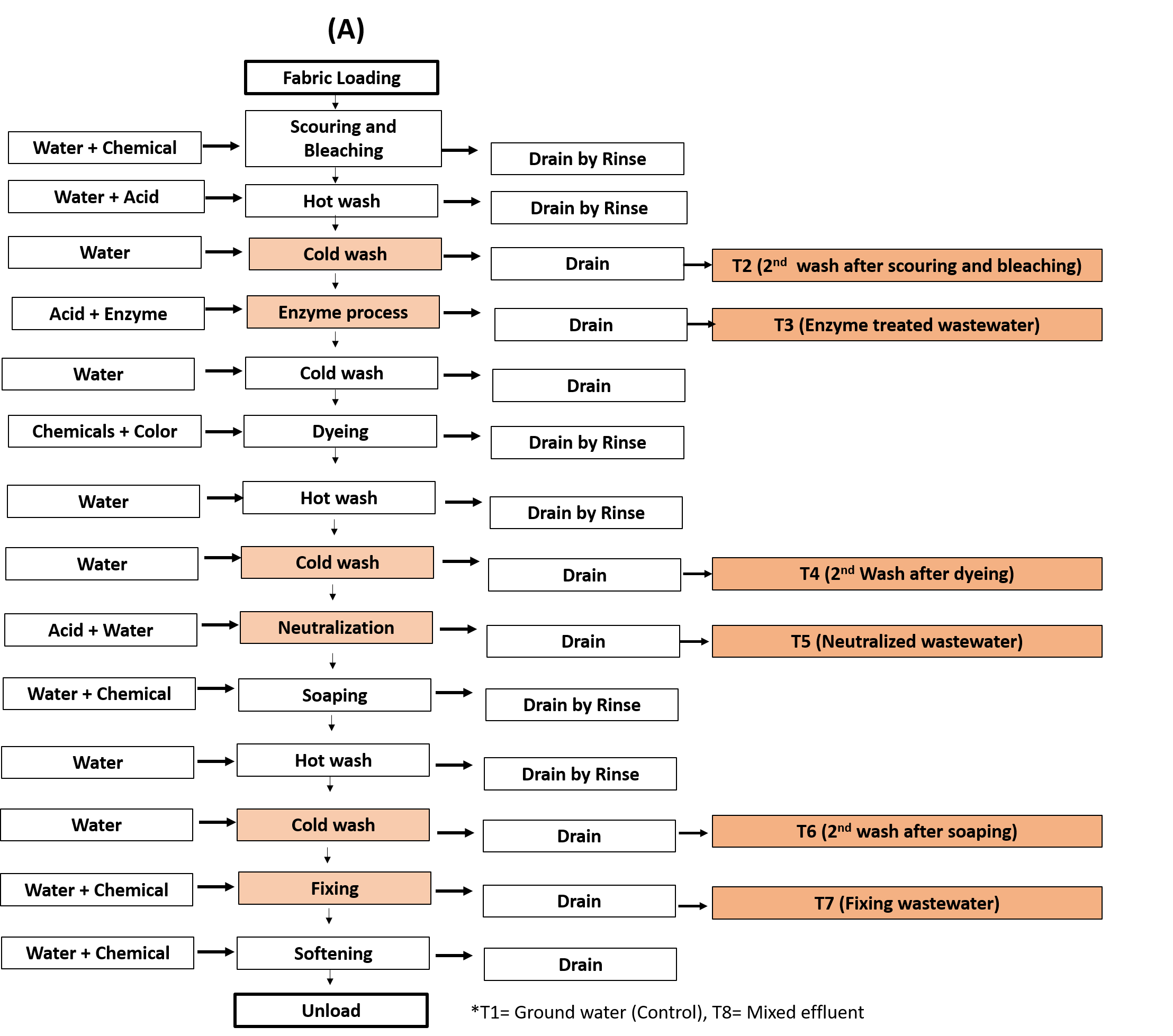


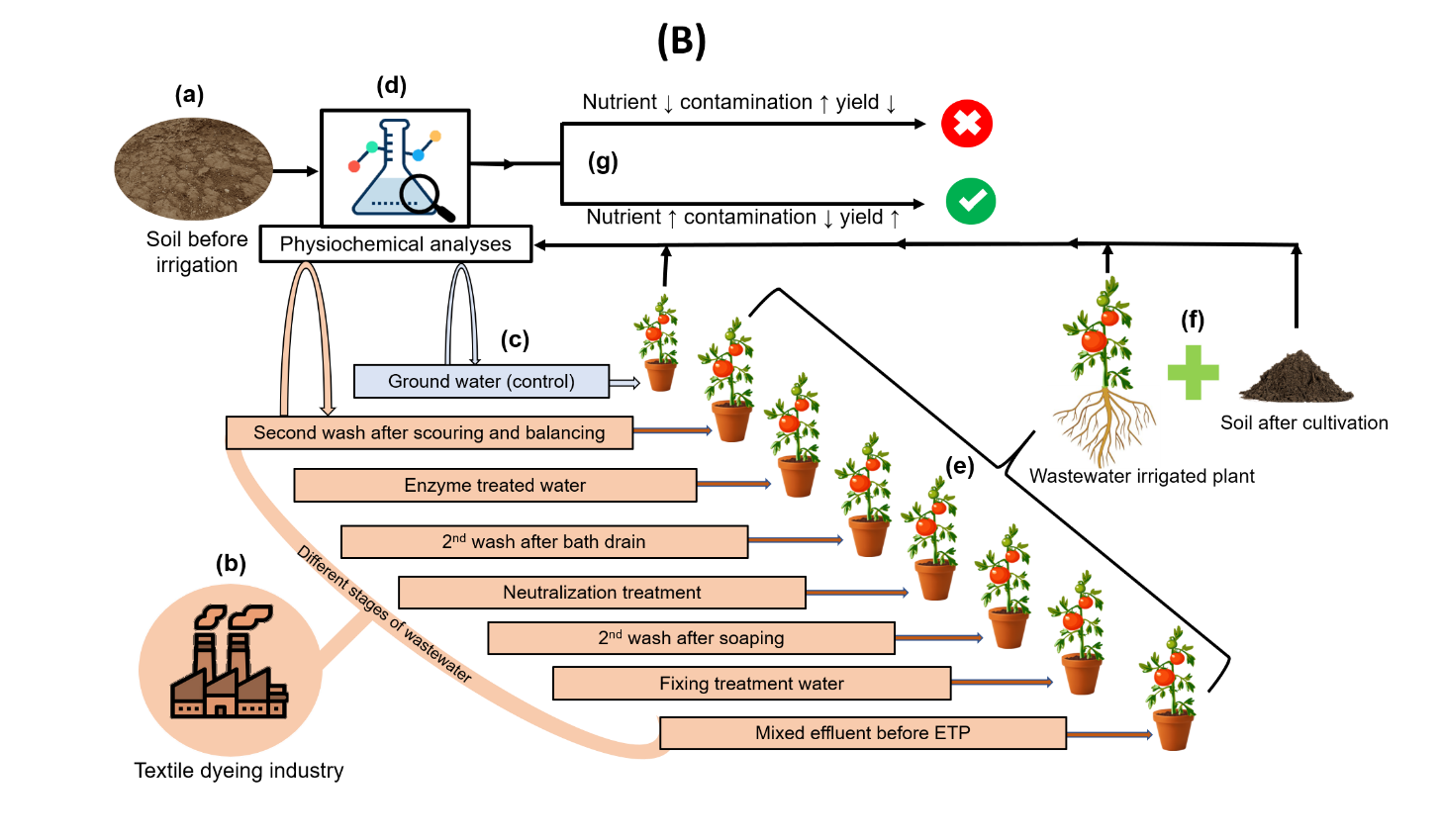


**Supplementary Fig.1.** Hypothetical illustration of optimizing different stages of textile dyeing wastewater (TDW) for tomato cultivation. (A) Flow diagram of different stages in a dyeing process of a textile industry; Color shaded box indicates the wastewater sample collection stages. (B) Feasibility of TDW for tomato irrigation. (a) Fresh soil before irrigation; (b) Different stages of TDW collected from (A); (c) Groundwater (GW) as control; (d) Physiological attributes of soil, TDW, GW; (e) Tomato growth and yield irrigated with TDW, GW; (f) Pollution load index (PLI) in irrigated soil after harvest; (g) Hypothesis evaluation whether TDW has impact on tomato growth, yield and soil properties.
